# Peptide mini-scaffold facilitates JNK3 activation in cells

**DOI:** 10.1038/srep21025

**Published:** 2016-02-12

**Authors:** Xuanzhi Zhan, Henriette Stoy, Tamer S. Kaoud, Nicole A. Perry, Qiuyan Chen, Alejandro Perez, Sylvia Els-Heindl, Jack V. Slagis, Tina M. Iverson, Annette G. Beck-Sickinger, Eugenia V. Gurevich, Kevin N. Dalby, Vsevolod V. Gurevich

**Affiliations:** 1 Departments of Pharmacology, Vanderbilt University, Nashville, TN 37232, USA; 2 University of Tübingen, Tübingen 72074, Germany; 3 Faculty of Pharmacy, Minia University, Minia, Egypt; 4 Division of Medicinal Chemistry, The University of Texas at Austin, Austin, Texas 78712, USA; 5 Universität Leipzig, Faculty of Biosciences, Pharmacy and Psychology, Institute of Biochemistry, Brüderstrasse 34, 04103 Leipzig, Germany; 6 Departments of Biochemistry, Vanderbilt University, Nashville, TN 37232, USA; 7 Center for Structural Biology, Vanderbilt University, Nashville, TN 37232, USA; 8 Vanderbilt Institute of Chemical Biology, Vanderbilt University, Nashville, TN 37232, USA

## Abstract

Three-kinase mitogen-activated protein kinase (MAPK) signaling cascades are present in virtually all eukaryotic cells. MAPK cascades are organized by scaffold proteins, which assemble cognate kinases into productive signaling complexes. Arrestin-3 facilitates JNK activation in cells, and a short 25-residue arrestin-3 peptide was identified as the critical JNK3-binding element. Here we demonstrate that this peptide also binds MKK4, MKK7, and ASK1, which are upstream JNK3-activating kinases. This peptide is sufficient to enhance JNK3 activity in cells. A homologous arrestin-2 peptide, which differs only in four positions, binds MKK4, but not MKK7 or JNK3, and is ineffective in cells at enhancing activation of JNK3. The arrestin-3 peptide is the smallest MAPK scaffold known. This peptide or its mimics can regulate MAPKs, affecting cellular decisions to live or die.

The spatial and temporal organization of proteins within a cell is critical for coordinating essential activities^1^. Appropriate cellular response to external or internal stimuli often requires precise orchestration by scaffold proteins, which determine the specificity and precise time course of signaling. In particular, the specificity of signal transduction through mitogen activated protein kinase (MAPK) cascades is highly dependent on scaffold proteins^2^,^3^,^4^. MAPK signaling is involved in the regulation of key cellular behaviors, from proliferation to differentiation and apoptotic death^4^. The overall architecture of three-kinase MAPK cascades is conserved from yeast to mammals^5^. Most cells have multiple MAPKs, MAPK kinases (MAPKKs), and MAPKK kinases (MAPKKKs), so signaling outcome is often determined by scaffolds organizing particular MAPKKK-MAPKK-MAPK complexes^2^,^3^,^4^,^6^.

The c-Jun NH_2_-terminal protein kinases (JNKs) belong to the MAPK family. JNKs regulate normal physiological processes of cell proliferation, apoptosis, differentiation, and migration^7^. JNKs were also implicated in many diseases, from cancer to neurological and immunological disorders^8^,^9^,^10^. Full activation of all JNKs requires double phosphorylation of the T-X-Y motif in the activation loop by two upstream kinases, MKK4 (tyrosine) and MKK7 (threonine)^11^. Similar to other MAPKs, JNK activation is dependent on scaffolding proteins, such as JIPs^12^. Arrestins, which specifically bind active phosphorylated G protein-coupled receptors (GPCRs), were first discovered as negative regulators of GPCR signaling via G proteins^13^,^14^. Among the four arrestin subtypes expressed in vertebrates^15^, only arrestin-3 promotes the activation of JNK3^16^, as well as ubiquitous JNK1/2^17^ in cells, acting as a scaffold that brings together MAPKKK ASK1^16^,^18^, MAPKKs MKK4^16^,^18^,^19^ and MKK7^17^,^20^, and several isoforms of JNK1/2/3^16^,^17^,^18^,^21^,^22^. Recently we identified the first 25 residues of arrestin-3 as the key JNK3 binding site^23^. Here we demonstrate that this short arrestin-3-derived peptide also binds ASK1 and MKK4/7 and facilitates JNK3 activation in intact cells. This is the smallest JNK cascade scaffold discovered so far. Its size paves the way to designing small molecule mimics that can be used as tools for targeted manipulation of anti-proliferative and often pro-apoptotic JNK signaling in cells.

## Results and Discussion

We recently found that while three elements in both arrestin-3 domains are involved in JNK3 binding, the peptide representing the first 25 residues of arrestin-3 (T1A) is the key interaction site^23^. This opens up three possibilities. First, if T1A only binds JNK3, but not the other kinases in the cascade, it could recruit JNK3 away from functional scaffolds, thereby suppressing JNK3 activation. Second, if T1A binds several kinases in the JNK3 activation module, but does not promote JNK3 phosphorylation, it might act as a dominant-negative silent scaffold, similar to arrestin-3-KNC mutant we recently described^24^. Finally, if T1A binds the same kinases as arrestin-3 and facilitates the signaling in the JNK3 cascade, it would be the smallest active MAPK scaffold known, which opens new avenues for the manipulation of MAPK signaling in cells for research and therapeutic purposes.

**Figure 1:**
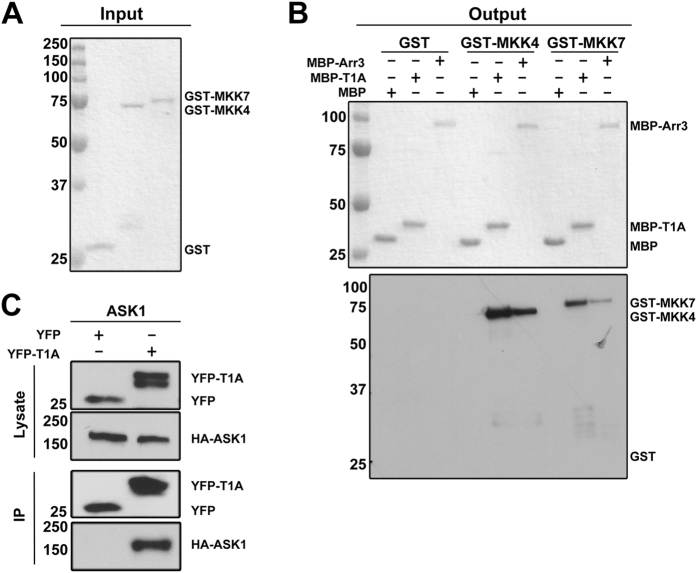
The T1A peptide binds ASK1, MKK4, and MKK7. (**A**) Purified GST (control), GST-MKK4, and GST-MKK7 (Coomassie staining) (**B**) Top: Coomassie staining of MBP (control), MBP-T1A, and MBP-arrestin-3 bait eluted from amylose beads. Lower blot: GST-MKK4 and GST-MKK7 retained by MBP-T1A and MBP-arrestin-3. Pull-down was performed as described in methods. (**C**) HA-ASK1 was co-immunoprecipitated with YFP-T1A, but not the YFP control, from cells coexpressing these proteins (top blots: lysate; bottom blots: proteins immunoprecipitated with anti-GFP antibody). Full blots are shown in Supplemental Fig. S1.

To determine the functional capabilities of T1A peptide we took advantage of the availability of purified MKK4 and MKK7, both of which activate JNK3^11^ and were shown to bind full-length arrestin-3^20^. We expressed T1A in *E. coli* as an MBP-fusion and purified it on an amylose column^23^. The ability of purified GST-MKK4 or GST-MKK7 (Fig. 1A) to bind MBP-T1A immobilized on an amylose column was tested in an *in vitro* pull-down assay, where MBP and MBP-arrestin-3 served as negative and positive controls, respectively (Fig. 1B). MBP-T1A, but not control MBP, effectively retained both kinases (Fig. 1B). Interestingly, similar to full-length arrestin-3, T1A peptide demonstrated stronger interaction with MKK4 than with MKK7 (Fig. 1B, lower panel). Thus, in addition to JNK3^23^, T1A peptide binds both MKKs known to phosphorylate it. Since the pull-down was performed with purified proteins, the data prove that the interactions of T1A with MKK4 and MKK7 are direct and do not involve any intermediaries or helpers. Next, we tested whether T1A binds the uppermost kinase in the cascade, ASK1. Because ASK1 is not available in purified form, we co-expressed HA-tagged ASK1 and YFP-tagged T1A (using YFP as a control) in COS7 cells, lysed the cells, and immunoprecipitated YFP constructs with anti-GFP antibody (Fig. 1C). We found that HA-ASK1 was effectively co-immunoprecipitated with YFP-T1A, but not with control YFP (Fig. 1C). Thus, in addition to JNK3^23^, T1A peptide binds all upstream kinases of its activation cascade, ASK1, MKK4, and MKK7 (Fig.1).

**Figure 2:**
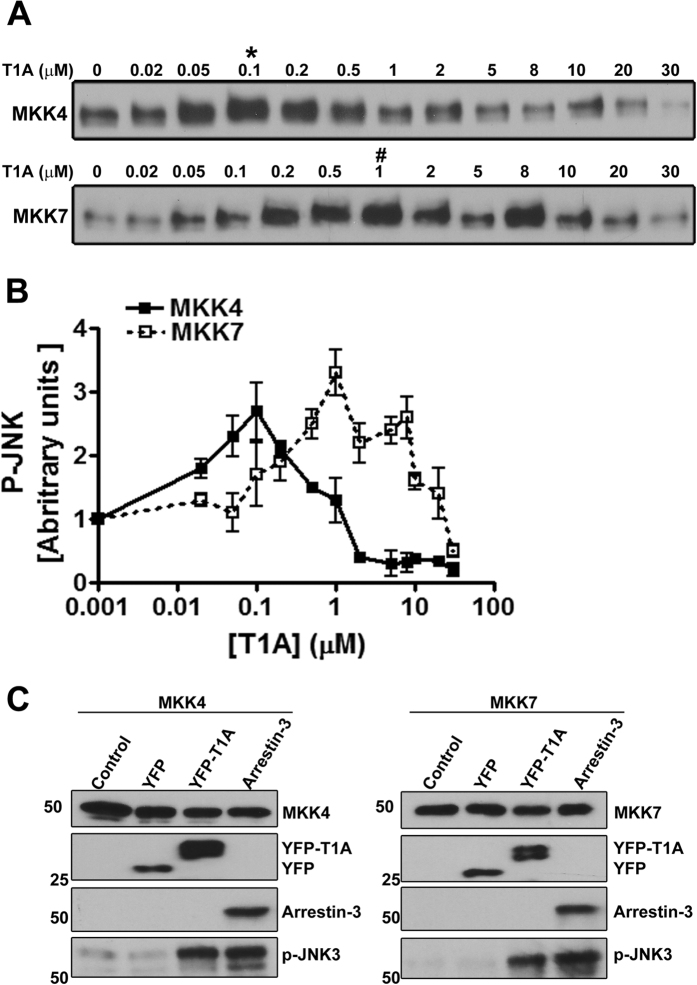
T1A facilitates JNK3 phosphorylation by MKK4 and MKK7. (**A**) Representative autoradiograms showing JNK3α2 phosphorylated by purified MKK4 (upper panel) and MKK7(lower panel) at the indicated concentration of synthetic purified T1A peptide (10-s incubation). (**B**) Quantification of JNK3α2 phosphorylation by MKK4 and MKK7. (**C**) JNK3α2 phosphorylation by MKK4 and MKK7 in COS7 cells co-expressing JNK3α2 with MKK4 or MKK7 (control) and YFP, YFP-T1A, or arrestin-3. Full blots are shown in Supplemental Fig. S2.

Next, we tested whether T1A acts as a scaffold facilitating signaling. To this end, we used experiments with purified proteins, because they provide the most definitive data supporting a direct interaction. As purified ASK1 is not available, we reconstituted MKK4-JNK3 and MKK7-JNK3 modules in the absence and presence of varying concentrations of synthetic purified T1A peptide and measured JNK3 phosphorylation (Fig. 2A). In both cases we obtained bell-shaped curves reflecting JNK3 phosphorylation level as a function of T1A concentration (Fig. 2B). This dependence, where signaling is increased at lower scaffold concentrations and decreased at higher, was also found for scaffolding of these modules by full-length arrestin-3^20^. It is believed to be a characteristic of simple scaffolds, which act by bringing the enzyme and substrate together, as lower scaffold concentrations make the formation of complete scaffold-enzyme-substrate ternary complexes likely, whereas higher concentrations increase the probability of formation of incomplete enzyme-scaffold and substrate-scaffold binary complexes, suppressing the signaling^25^,^26^. The optimal concentration of a scaffolding protein for signaling depends on its affinity for the proteins it scaffolds^20^,^25^,^26^.

Our previous work suggested that the efficiency of JNK3 phosphorylation in the complex that includes MKK4 and arrestin-3 is higher than the efficiency of JNK3 phosphorylation by MKK4 in the absence of arrestin-3^20^. Since these experiments were performed with pure components, the data show that simultaneous direct binding to T1A of MKK4/7 (Fig. 1A) and their substrate JNK3^23^ places MKKs into a favorable position to phosphorylate JNK3 (Fig. 2A,B). Previously we found that the optimal concentrations of arrestin-3 for scaffolding MKK4-JNK3 and MKK7-JNK3 modules are ~0.6 μM and ~ 6 μM^20^, reflecting lower affinity of arrestin-3 for MKK7 than for MKK4^20^. Interestingly, the optimal concentration of T1A for promoting JNK3 phosphorylation by MKK4 is lower than for MKK7-JNK3 module (Fig. 2B), in line with more avid binding of this peptide to MKK4 (Fig. 1B). Intriguingly, in both cases optimal T1A concentrations were ~10-times lower than those of full-length arrestin-3. These results suggest that T1A has higher affinity for MKKs and/or JNK3, in line with its ability to retain more MKKs than arrestin-3 (Fig. 1B). This phenomenon might be explained by greater accessibility of T1A peptide when it is free and not in the context of full-length arrestin-3, where it is partially shielded by other elements of the N-domain^27^.

To test whether T1A can promote JNK3 phosphorylation by MKKs in cells, we co-expressed MKK4 or MKK7 with YFP-T1A in COS7 cells, using YFP and full-length arrestin-3 as negative and positive controls, respectively (Fig. 2C). We found that YFP-T1A, but not YFP, increases JNK3 phosphorylation by both MKKs, similar to arrestin-3. Thus, T1A is necessary and sufficient to promote the signaling in MKK4/7-JNK3 modules both *in vitro* and in intact cells.

**Figure 3:**
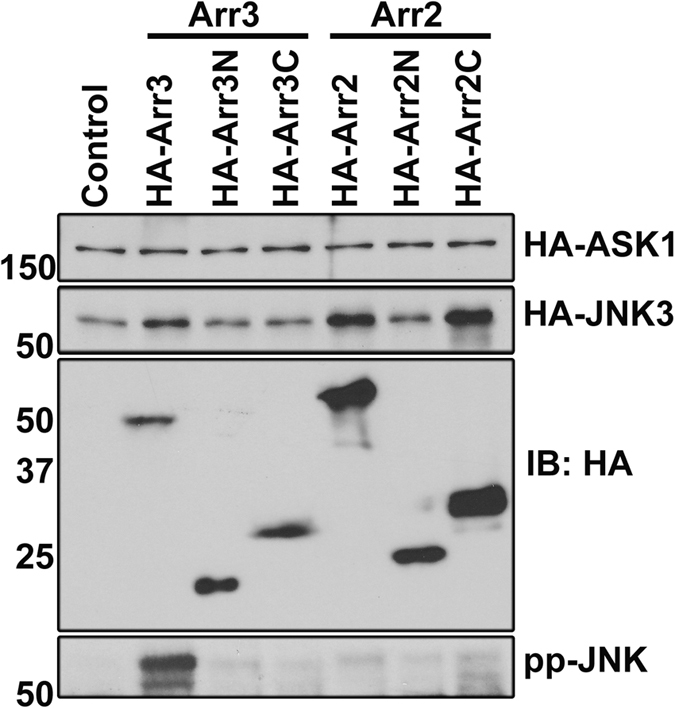
Separated arrestin domains do not promote JNK activation. Western blot of lysates of COS7 cells co-expressing HA-ASK1 and HA-JNK3α2 (Control), or with HA-tagged full-length arrestin-3 (Arr3), arrestin-2 (Arr2), or separated N- and C-domains of the two non-visual arrestins (Arr3N, Arr2N, Arr3C, Arr2C). Phospho-JNK blot shows that among these constructs only full-length arrestin-3 facilitates JNK3α2 phosphorylation in cells. Full blots are shown in Supplemental Fig. S3.

T1A constitutes a small part of the arrestin-3 N-domain^27^. All arrestins consist of two domains^27^,^28^,^29^,^30^, which fold independently, can be expressed separately, and retain certain functions^18^,^31^,^32^. Therefore, we tested whether separated domains of arrestin-3, as well as the other ubiquitously expressed non-visual subtype, arrestin-2, which also binds kinases of the JNK3 activation cascade^18^, can promote JNK3 phosphorylation in cells. To this end, we expressed in COS7 cells both arrestins and their individual domains with the same HA-tag (to compare their expression on the same blot) along with ASK1 and JNK3 (Fig. 3). We confirmed that arrestin-3 expression significantly increases JNK3 phosphorylation in cells, whereas arrestin-2 does not^16^,^18^,^33^, and found no effect of the separated domains of either arrestin (Fig. 3).

**Figure 4:**
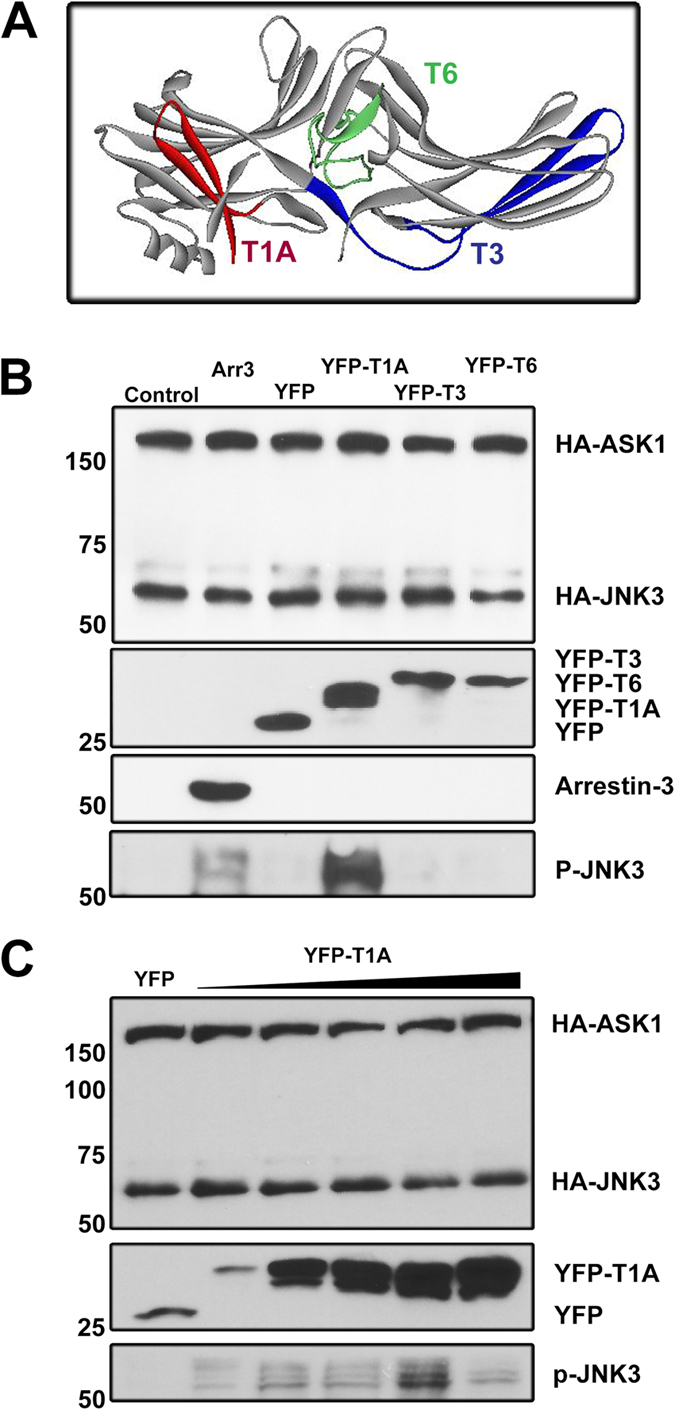
T1A facilitates JNK3 activation in cells. (**A**) The structure of arrestin-3 (PDB: 3P2D;^27^) with the three peptides implicated in JNK3 binding^23^ shown in red (T1A), blue (T3), and green (T6). (**B**) COS7 cells co-expressed HA-ASK1 and HA-JNK3α2 without (Control) or with full-length arrestin-3 (Arr3), YFP, or indicated YFP-tagged JNK3-binding peptides. The upper three blots show expression levels of indicated proteins; the lower blot shows that T1A facilitates JNK3α2 phosphorylation more efficiently than full-length arrestin-3. (**C**) COS7 cells co-expressed HA-ASK1 and HA-JNK3α2 with YFP (Control) or different concentrations of YFP-T1A. The upper two blots show expression levels of indicated proteins; the lower blot shows biphasic dependence of JNK3α2 phosphorylation on T1A level. Full blots are shown in Supplemental Fig. S4b (panel B) and S4c (panel C).

T1A effectively enhanced JNK3 phosphorylation by MKK4 and MKK7 (Figs 1,2), whereas full-length arrestin-3 facilitates JNK3 phosphorylation upon co-expression with ASK1^16^,^18^,^21^,^33^, which phosphorylates and activates MKKs. If the arrestin-3 N-domain fails to promote the activation of JNK3 because it can only scaffold MKK4/7-JNK3 modules, then the T1A peptide, which constitutes only a part of the arrestin-3 N-domain, would not be expected to promote JNK3 phosphorylation in the presence of ASK1 in cells. To test this, we co-expressed YFP fusions of T1A and two other JNK3-binding peptides T3 and T6 (Fig. 4A)^23^, with JNK3 and ASK1 in COS7 cells and monitored JNK3 phosphorylation, using YFP and arrestin-3 as negative and positive controls, respectively (Fig. 4B). As expected, arrestin-3 increased the level of JNK3 phosphorylation (Fig. 4B). T1A, but not other peptides, was also active, and the effect of T1A was greater than that of full-length arrestin-3 (Fig. 4B). To test whether T1A still functions as a simple scaffold in intact cells in the presence of over-expressed ASK1, as in case of MKK4/7-JNK3 signaling modules (Fig. 2), we co-expressed ASK1 and JNK3 with YFP (control) and varying amounts of YFP-T1A (Fig. 4C). The dose-response curve for T1A in these experiments was also biphasic, with a clear optimum, indicating that T1A is a simple scaffold of the three-kinase cascade, similar to full-length arrestin-3^19^.

**Figure 5:**
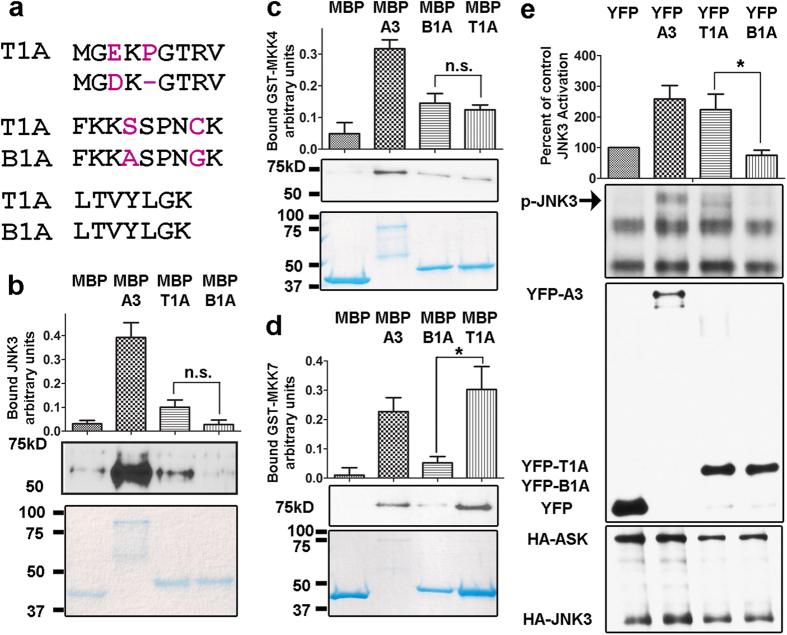
T1A activity is specific. (**a**) Sequence comparison of arrestin-3-derived T1A and B1A, derived from arestin-2, which does not facilitate JNK3 activation . Residues that differ between T1A and B1A are shown in magenta. (**b–d**) Pull-down of purified JNK3 (**b**), MKK4 (**c**), and MKK7 (**d**) by MBP (negative control), MBP-arrestin-3 (positive control), MBP-T1A, and MBP-B1A, was performed, as described in Methods. Lower panels, Coomassie gels of loaded MBP-fusions; middle panels, Western blots of retained JNK3 (**b**), MKK4 (**c**), and MKK7 (**d**); upper panels, quantification of Western blots from 3-4 independent experiments. Note that B1A binds MKK4 like T1A, but does not appreciably interact with JNK3 or MKK7. (**e**) COS7 cells co-expressed HA-ASK1 and HA-JNK3α2 with YFP (negative control), YFP-arrestin-3 (Arr3, positive control), YFP-T1A, or YFP-B1A. Lower two blots show expression levels of indicated proteins; upper blot and bar graph (quanitification of JNK3α2 phosphorylation in four independent experiments) show that T1A facilitates JNK3α2 phosphorylation, whereas B1A does not. *p < 0.05; n.s., not significant. Full blots and gels are shown in Supplemental Fig. S5.

To test the specificity of T1A action, we compared its ability to bind purified MKK4, MKK7, and JNK3 with that of B1A, a homologous N-terminal peptide from closely related arrestin-2, which differs from T1A only in four positions (Fig. 5). We chose B1A as a “natural” negative control, because arrestin-2, despite 78% sequence identity to arrestin-3^34^, does not promote JNK3 activation in cells^16^,^18^,^21^. We found that while both peptides comparably bind MKK4, B1A demonstrates less robust interaction with JNK3 and its other upstream activator, MKK7 (Fig. 5). To test whether this difference in binding translates into differential activity in cells, we compared the ability of YFP-T1A and YFP-B1A to facilitate JNK3 activation in cells over-expressing ASK1, using YFP-arrestin-3 and YFP as positive and negative controls, respectively. We found that in contrast to T1A, arrestin-2-derived B1A does not function as a scaffold of ASK1-MKK4/7-JNK3 cascade in cells (Fig. 5e), indicating that specific sequence, rather than simple accessibility of this part of arrestins, determines its functional capabilities.

Thus, the first 25 residues contained in the T1A peptide are primarily responsible for the ability of arrestin-3 to scaffold the ASK1-MKK4/7-JNK3 signaling module. The lack of the activity of the arrestin-3 N-domain (Fig. 3), which contains the T1A peptide, along with facilitation of JNK3 phosphorylation in exactly the same experimental paradigm by T1A (Fig. 4), clearly indicates that when separated, this peptide is a lot more accessible than in the context of arrestin-3 or its N-domain. It is tempting to speculate that receptor binding of arrestin-3 stimulates its ability to promote JNK3 activation^16^ by increasing the accessibility of the T1A element to relevant kinases. Significant flexibility of receptor-bound arrestins revealed by biophysical methods^35^,^36^ supports this idea. While the crystal structure of arrestin-3 in complex with any GPCR is not available, the only existing structure of the arrestin-receptor complex, that of visual arrestin-1 bound to rhodopsin^37^, is consistent with this hypothesis. Arrestin-3 elements that promote signaling leading to the activation of other MAPKs, such as ERK1/2^38^ and p38^39^, also need to be identified. The activation of these two kinases is strictly dependent on arrestin binding to the receptor^38^,^39^, suggesting that arrestin elements that change conformation and/or become more exposed upon GPCR interaction are likely the prime suspects.

Our data identify a relatively short arrestin-3-derived peptide as an effective scaffold of the ASK1-MKK4/7-JNK3 signaling cascade, making it the smallest MAPK scaffold ever reported. However, T1A can be large enough to bind all the kinases involved simultaneously. Each residue has a structure –NH-CH(R)-CO-, with each bond >1.4A. Thus, taking into account angles, a single reside has a length of ~3A. Therefore, a 25-residue peptide in fully extended conformation can achieve a length of up to ~75A, which is similar to the maximum “wingspan” (the distance between the far tips of the two arrestin domains) of all arrestins^27^,^28^,^29^,^30^. Nonetheless, limited size of T1A opens the prospect of designing peptides and/or non-peptide small molecule mimics that can be used as tools to manipulate MAPK signaling for research and therapy. Many human disorders are caused by excessive cell proliferation (e.g., cancer) or death (e.g., Alzheimer’s, Parkinson’s, and other neurodegenerative diseases). Targeted activation of JNK family kinases usually has anti-proliferative effect, whereas the activation of ERK1/2 promotes cell survival. Scaffolds facilitating signaling in these pathways might be easier to dose than direct pharmacological activators, making them safer intervention tools. The identification of the shortest active form of the T1A-derived peptide must be the next step in this direction.

## Methods

### Materials

All restriction and DNA modifying enzymes (T4 DNA ligase, Vent DNA polymerase, and calf intestine alkaline phosphatase) were from New England Biolabs (Ipswich, MA). Other chemicals were from sources recently described^17^,^23^.

### Terminology

We use the systematic names of arrestin proteins: arrestin-1 (historic names S-antigen, 48 kDa protein, visual or rod arrestin), arrestin-2 (β-arrestin or β-arrestin1), arrestin-3 (β-arrestin2 or hTHY-ARRX), and arrestin-4 (cone or X-arrestin; for unclear reasons its gene is called “*arrestin 3*” in the HUGO database).

### MBP-fusion protein constructs in pMal and MBP pull-down

To make MBP-fusions containing arrestin-2/3 elements, the cDNAs encoding arrestin fragments were subcloned into pMal-p2T (generous gift from Dr. Keiji Tanaka, Tokyo Institute of Medical Science) between Eco RI and Xho I sites in frame with MBP, as described^23^. MBP-Arr3 (full-length arrestin-3) was created by subcloning the corresponding cDNA into pMal-p2T between Eco RI and Not I sites^23^. All MBP-arrestin-3 fusion proteins contained the same TLVPRGSPGF linker between MBP and arrestin-3 or its fragments. The MBP protein used as negative control was purified using empty pMal-p2T vector containing the same linker with 10 additional residues: PGRLERPHRD. MBP fusions were purified, as described^23^. MBP pull-down was performed, as described^23^. Briefly, Indicated MBP fusions (10-30 μg in 50 μL 20 mM Tris/150 mM NaCl) were immobilized on amylose resin (25 μL, 50% slurry, New England Biolabs) for 1 h at 4 °C with slight rotation. Purified, as described^40^, MKK4/7 (10 μg in 50 μL 20 mM Tris/150 mM NaCl) were added to the immobilized MBP fusions and rotated gently for 2 h at 4 °C. Samples were transferred to centrifuge filters (Durapore^®^-PVDF-0.65 μm), washed three times with 50 mM HEPES-Na, pH 7.3, 150 mM NaCl. The proteins were eluted by 100 μl of elution buffer (wash buffer containing 50 mM maltose) by gentle rotation for 5 min at 4 °C. Eluates were analyzed by SDS-PAGE and Western blotting.

### Kinase purification and *in vitro* phosphorylation assay

JNK3a2^20^,^23^, and MKK4 and MKK7^17^,^20^ were expressed in *E. coli* and purified as previously described^40^. The effect of arrestin-3 and MBP-T1A on the phosphorylation of JNK3α2 by MKK7 or MKK4 was analyzed by an *in vitro* kinase assay, as described^19^,^20^. Briefly, the assays were conducted in 10 μL containing the following final concentrations: 50 nM active MKK7 or MKK4, 1 μM JNK3α2, and indicated concentrations of arrestin-3 or T1A. The mixtures were incubated individually at 30 °C for 10 sec. The reactions were stopped by the addition of 15 μL of Laemmli SDS sample buffer (Sigma) and 2 μl of total reaction sample was subjected to SDS-PAGE (8%) and transferred polyvinylidene difluoride (PVDF) membranes (Millipore, Bedford, MA). Phosphorylated JNK3α2 was visualized by rabbit anti-phospho JNK antibody (Cell Signaling) and the level of JNK phosphorylation was quantified.

### Peptide synthesis

The T1A peptide (MGEKPGTRVFKKSSPNCKLTVYLGK), representing the first 25 residues of arrestin-3, was synthesized using automated synthesis robot (SyroI, MultiSyntech, Witten, Germany) on NovaSyn^®^ TGR R resin (13.5 μmol, Novabiochem, Darmstadt, Germany) with a fluorenylmethyloxycarbonyl chloride (FMOC)/tert-butyl strategy, as described^41^. FMOC-amino acids were from Iris Biotech and Novabiochem (Marktredwitz, Germany). Amino acid side chain protecting groups were used as following: trityl (Trt) for Asn and Cys; tBu for Glu, Thr, Ser and Tyr; tert-butyloxycarbonyl (Boc) for Lys; and pentamethyl-2,3-dihydrobenzofuran-5-sufonyl (Pbf) for Arg. Automated FMOC deprotection was carried out with 40% (v/v) piperidine (Sigma-Aldrich, Taufkirchen, Germany) in N,N-dimethylformamide (DMF; Biosolve, Valkenswaard, The Netherlands) for 3 min and 20% (v/v) piperidine in DMF for 10 min. The coupling of the amino acids was carried out twice. FMOC-amino acids were pre-incubated with OxymaPure (Iris Biotech) for 2 min. Following the addition of N,N’-diisopropylcarbodiimide (DIC; Iris Biotech), reaction was incubated for 40 min. After successful synthesis, peptides were cleaved from the resin with 90% trifluoroacetic acid (TFA) and 10% 1,2-ethanedithiol/thioanisole (v/v 3:7). Methionines were reduced with 1,2-ethanedithiol and trimethylsilyl bromide in TFA. Subsequently, peptides were purified on a reversed-phase C18 column (Phenomenex Jupiter 10u Proteo 90 Å: 250 × 21.2 mm; 7.8 μm; 90 Å) and analyzed by MALDI-TOF mass spectrometry (UltraflexII, Bruker, Bremen, Germany) and analytical reversed-phase HPLC on columns Phenomenex Kinetex 5u XB-C18 100 Å (Phenomenex: 250 × 4.6 mm; 5 μm; 100 Å) and Phenomenex Jupiter 4u Proteo 90 Å (Phenomenex: 250 × 4.6 mm; 4 μm; 90 Å). Eluent A was 0.1% TFA in H_2_O and eluent B 0.08% TFA in ACN. On both columns, a gradient of 10% eluent B in A to 60% eluent B in A in 40 min was used. Following this procedure, the peptide was ≥95% pure (theoretical M_r_ = 2766.5 Da, experimental ([M + H]^+^) = 2767.6).

### Plasmids, Cell Culture, and Transient Transfection

HA-tagged arrestins and their separated domains were constructed, as described^31^. Full-length arrestin-3 and its fragments with N-terminal YFP tag were constructed by subcloning the cDNA encoding YFP-arrestin-3 or YFP-tagged arrestin-3 fragments cDNAs into pcDNA3.1 between Eco RI and Hind III restriction sites.

COS7 African green monkey cells were maintained in DMEM supplemented with 10% heat-inactivated FBS (Invitrogen), penicillin, and streptomycin at 37 °C in a humidified incubator with 5% CO_2_. The cells were plated at 80–90% confluence and transfected using Lipofectamine 2000 (Invitrogen) according to the manufacturer’s instructions. Cells were used 48 h post-transfection and serum-starved overnight before experiments.

### Western Blotting and measurement of JNK phosphorylation in intact cells

COS7 cells were incubated with phosphatase inhibitors (50 mM NaF and 10 mM Na_3_VO_4_) in serum-free medium for 15 min at 37 °C; washed with cold PBS; and lysed with SDS lysis buffer containing 1% SDS, 10 mM Tris (pH7.4), 10 mM NaF, 100 μM Na_3_VO_4_, 2 mM EDTA, 2 mM benzamidine and 1 mM PMSF. JNKs activity was assayed by Western blotting using an antibody specific for phosphorylated JNK to detect phophorylated (active) JNKs^17^,^20^,^23^. Whole cell lysates were boiled for 5 min and centrifuged at 10,000 × *g* for 10 min, and the supernatants were used for Western blotting. Protein was measured using the Bio-Rad Coomassie Blue assay. The proteins were resolved on 8% SDS-PAGE and transferred to PVDF membrane (Millipore, Bedford, MA). Blots were incubated with the primary antibodies (Cell Signaling Technology, Inc) anti-phospho-JNK, anti-JNK, anti-HA (6E2) (1:1000 to 1:5000), followed by appropriate HRP-conjugated secondary antibodies. Protein bands were detected by enhanced chemiluminescence (ECL, Pierce), followed by exposure to x-ray film. To quantify phopho-JNKs, we used serial dilutions of anisomycin (1 μg/ml)-stimulated HEK-A cell lysates to ensure that all samples were in linear range. The values for these proteins are expressed in arbitrary units.

## Additional Information

**How to cite this article**: Zhan, X. *et al*. Peptide mini-scaffold facilitates JNK3 activation in cells. *Sci. Rep*. **6**, 21025; doi: 10.1038/srep21025 (2016).

## Supplementary Material

Supplementary Information
