# Supplementary material for: Peptide mini-scaffold facilitates JNK3 activation in cells
Source: Sci Rep. 2016 Feb 12;6:21025. doi: 10.1038/srep21025 (PMC4751492; doi:10.1038/srep21025)
Supplement: Supplementary Information [file srep21025-s1.pdf]

Supplemental data
Peptide mini-scaffold facilitates JNK3 activation in cells

Xuanzhi Zhan, Henriette Stoy, Tamer S. Kaoud, Nicole A. Perry[1], Qiuyan Chen[1], Alejandro Perez, Sylvia Els-Heindl, Jack V. Slagis, Tina M. Iverson[1], Annette G. Beck-Sickinger, Eugenia V. Gurevich[1], Kevin N. Dalby, Vsevolod V. Gurevich

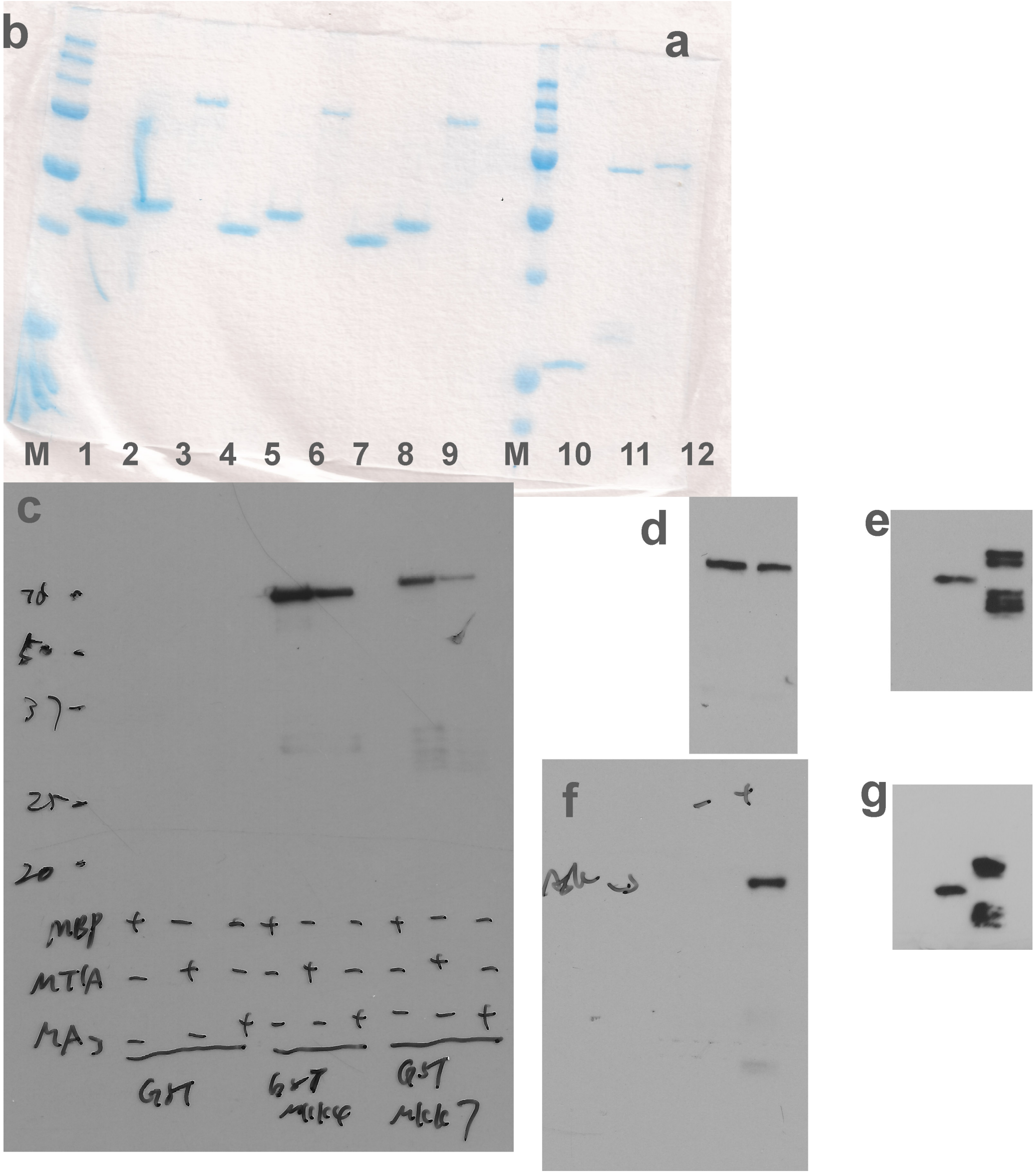

Fig. S1. T1A peptide binds ASK1, MKK4, and MKK7.

a Purified GST (control, lane 10), GST-MKK4 (lane 11), and GST-MKK7 (lane 12). Coomassie staining, the lane with molecular weight markers indicated as M.

b Coomassie staining of MBP (control, lanes 1, 4, 7), MBP-T1A (lanes 2, 5, 8), and MBP-arrestin-3 (lanes 3, 6, 9) bait eluted from amylose beads. Coomassie staining, the lane with molecular weight markers indicated as M.

c GST-MKK4 and GST-MKK7 retained by MBP-T1A and MBP-arrestin-3, but not MBP control (bait indicated under the blot). Pull-down was performed, as described in methods.

d-g HA-ASK1 was expressed at the same level (**d**) with YFP or YFP-T1A (**e**) and the YFP control and YFP-T1A were immunoprecipitated with anti-GFP antibody (**g**). HA-ASK1 co-immunoprecipitated with YFP-T1A, but not YFP control (**f**).

a

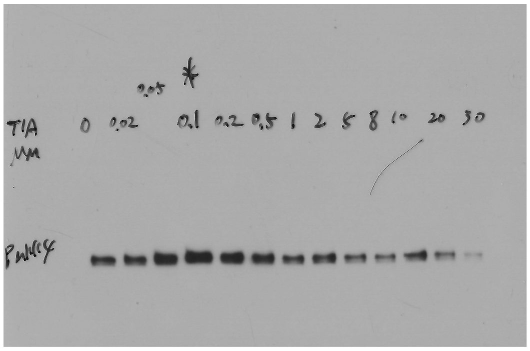

b

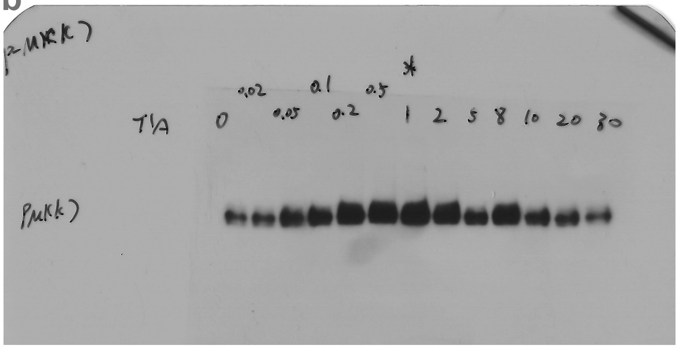

c

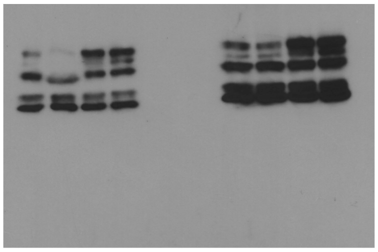

1 2 3 4 5 6 7 8

d

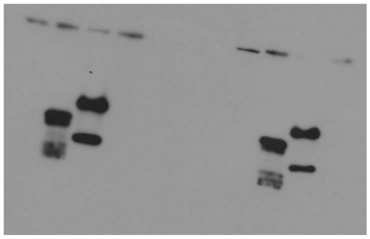

Fig. S2. T1A facilitates JNK3 phosphorylation by MKK4 and MKK7.

a Representative autoradiogram showing JNK3α2 phosphorylated by purified MKK4 at the indicated concentration of synthetic purified T1A peptide (10-s incubation).

b Representative autoradiogram showing JNK3α2 phosphorylated by purified MKK7 at the indicated concentration of synthetic purified T1A peptide (10-s incubation).

c JNK3α2 phosphorylation by MKK4 (lanes 1-4) and MKK7 (lasnes 5-8) in HEK293 cells co-expressing JNK3α2 with MKK4 or MKK7 (control, lanes 1, 5), YFP (lanes 2, 6), YFP-T1A (lanes 3, 7), or arrestin-3 (lanes 4, 8).

d The expression of YFP (lanes 2, 6) and YFP-T1A (lanes 3, 7) was detected by western blot with anti-GFP antibody, as described in methods.

Fig. S3. Separated arrestin domains do not promote JNK activation.

a,b Western blot of lysates of HEK293 cells co-expressing ASK1 with JNK3α2 (control, lane 1), or with HA-tagged full-length arrestin-3 (A3; lane 2), arrestin-2 (A2, lane 5), or separated N- and C-domains of the two non-visual arrestins (A3N, lane 3; A2N, lane 6; A3C, lane 4; A2C lane 7). Representative blots with anto-JNK3 antibody (**a**) and anti-HA antibody (**b**) are shown.

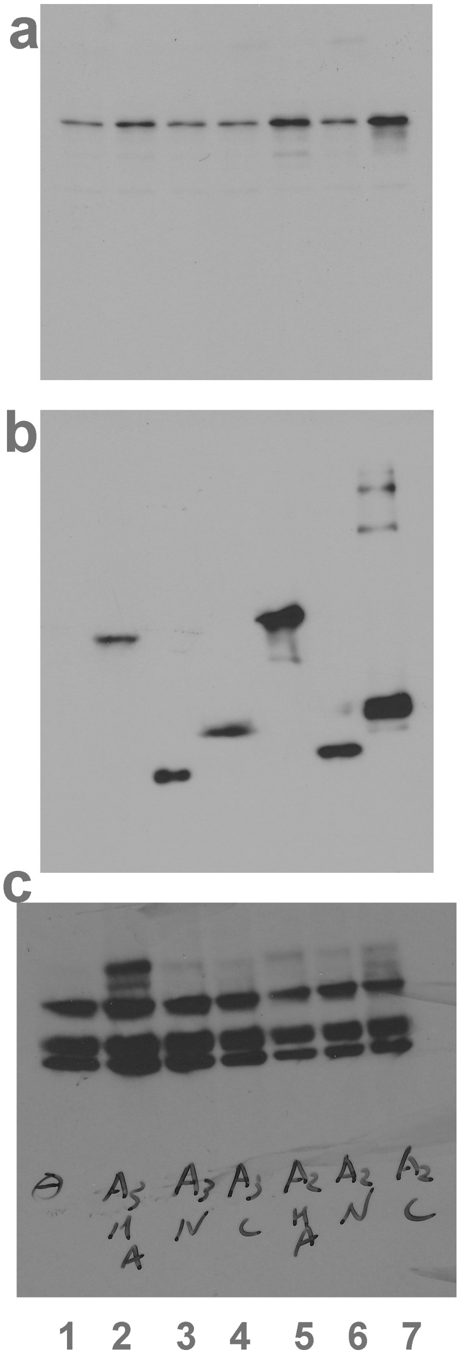

c Phospho-JNK blot shows that among these constructs only full-length arrestin-3 facilitates JNK3α2 (upper band) phosphorylation in cells. Three lower bands represent endogenous isoforms of JNK1 and JNK2 detected by the same pp-JNK antibody.

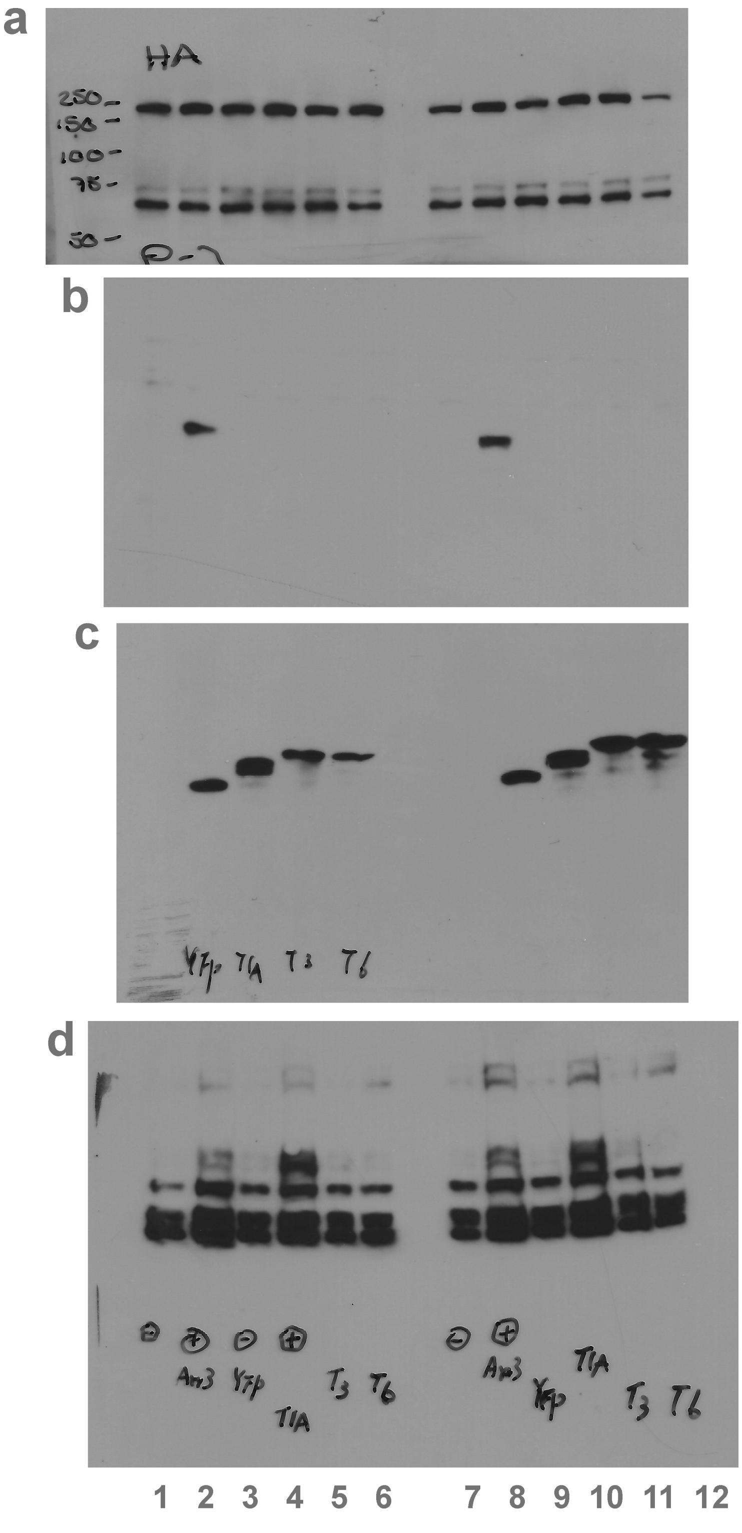

Fig. S4b. Full blots for Fig. 4b.

a-c HEK293 cells co-expressed HA-ASK1 and HA-JNK3α2 without (control, lanes 1, 7) or with full-length arrestin-3 (Arr3; lanes 2, 8), YFP (lanes 3, 9), or JNK3-binding peptides YFP-T1A (lanes 4, 10), YFP-T3 (lanes 5, 11), or YFP-T6 (lanes 6, 12) . Representative blots showing the expression levels of HA-ASK1 and HA-JNK3α2 (**a**), arrestin-3 (**b**), and YFP-containing constructs (**c**) are shown.

d Phospho-JNK blot shows that among these constructs only full-length arrestin-3 and YFP-T1A facilitate JNK3α2 (upper band) phosphorylation in cells. Three lower bands represent endogenous isoforms of JNK1 and JNK2 detected by the same pp-JNK antibody.

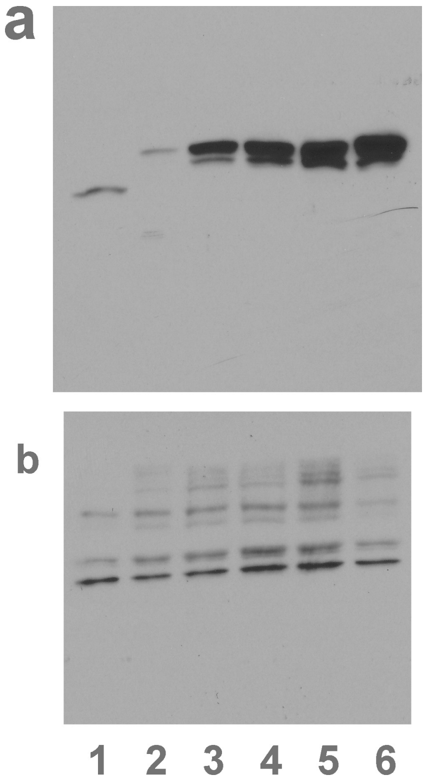

Fig. S4c. Full blots for Fig. 4c.

a HEK293 cells co-expressed HA-ASK1 and HA-JNK3α2 with YFP (control, lane 1) or different concentrations of YFP-T1A (lanes 2-6). Representative blot with anti-GFP antibody is shown.

b Biphasic dependence of JNK3α2 (upper band) phosphorylation on T1A level. Three lower bands represent endogenous isoforms of JNK1 and JNK2 detected by the same pp-JNK antibody.

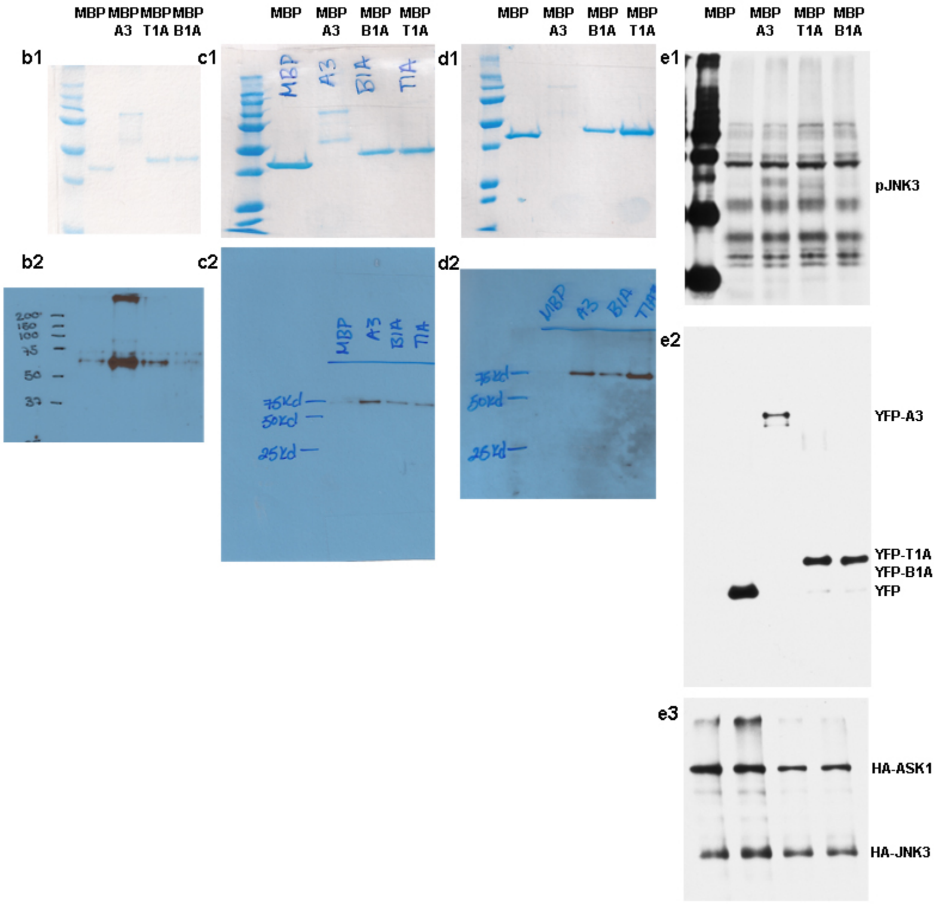

Fig. S5. T1A activity is specific.

b, c, d Pull-down of purified JNK3 (**b**), MKK4 (**c**), and MKK7 (**d**) by MBP (negative control), MBP-arrestin-3 (positive control), MBP-T1A, and MBP-B1A, was performed, as described in Methods. Upper panels (**b1, c1, d1**), full Coomassie gels of loaded MBP-fusions; lower panels (**b2, c2, d2**), full Western blots of retained JNK3 (**b**), MKK4 (**c**), and MKK7 (**d**).

e HEK293 cells co-expressed HA-ASK1 and HA-JNK3α2 with YFP (negative control), YFP-arrestin-3 (Arr3, positive control), YFP-T1A, or YFP-B1A. Lower blot (**e3**) shows expression of HA-ASK1 and HA-JNK3α2; middle blot (**e2**) shows the expression of YFP and YFP-tagged arrestin-3, T1A, and B1A; upper blot (**e1**) shows phsphorylated JNK3α2 (upper bands) and endogenous JNK1 and JNK2 (lower bands). The left lane shows biotinylated molecular weight markers (Bio-Rad) co-detected by avidin-peroxidase added along with secondary antibody.
